# Non-governmental organisations and the regulation of harmful commodity industries: navigating global governance to change corporate practices

**DOI:** 10.1186/s12992-026-01200-4

**Published:** 2026-03-13

**Authors:** Kathrin Lauber, Belinda Townsend, Liz Arnanz, Fran Baum, Katherine Cullerton, Sharon Friel, Rodney Holmes, Jane Martin, Rob Ralston, Jeff Collin

**Affiliations:** 1 School of Social and Political Science, University of Edinburgh, Edinburgh, UK; 2 School of Regulation and Global Governance, Australian National University, Canberra, Australia; 3 NCD Alliance, Geneva, Switzerland; 4 University of Adelaide, Stretton Institute, Adelaide, Australia; 5 School of Public Health, University of Queensland, Brisbane, Australia; 6 Foundation for Alcohol Research and Education (FARE), Canberra, Australia; 7 Food for Health Alliance, Melbourne, Australia

**Keywords:** Global health governance, Non-governmental organizations, Advocacy, Commercial determinants of health, Ultra-processed food, Alcohol

## Abstract

**Background:**

The crucial role of non-governmental organisations (NGOs) in advancing effective regulation of health-harming corporate practices is widely accepted, yet how precisely they shape global health governance is not well-understood. While a vast literature has grappled with corporate power and its political, market, and technological dimensions, less attention has been paid to strategic efforts to counter the influence of corporate actors. Drawing on international relations and political economy scholarship, we seek to understand NGO strategies aimed at achieving greater regulation of the ultra-processed food (UPF) and alcohol industries in global fora, and examine the considerations and constraints which inform NGO strategy choice.

**Methods:**

We conducted 22 semi-structured interviews with professional advocates active in global debates on UPF and/or alcohol governance, complemented by six interviews with officials from international organisations (IOs). Coding combined inductive and deductive approaches, with the latter informed by existing models of advocacy strategy.

**Results:**

Participants described ‘inside’ strategies targeting IOs and member states as the primary focus of their organisations’ global advocacy, underpinned by a sense that such approaches are more effective. More confrontational, outside approaches were seen as inappropriate particularly for targeting the World Health Organisation as a key forum. Core advocacy activities thus include participation in consultations and meetings hosted by IOs, although advocates did highlight less radical forms of ‘outside’ strategies, rooted in information provision rather than protest. The direct targeting of corporations was relatively uncommon, with monitoring and exposing of corporate practices primarily aimed at building momentum for governmental regulation. Advocates highlighted the importance of engagement beyond health governance, noting, however, that resource challenges preclude effective entry into new fora for many NGOs.

**Discussion:**

The challenge of attaining and maintaining insider status in relevant fora introduces constraints which may explain the reliance on quieter, less confrontational strategies. We reflect critically on the potential value of a noisier approach to addressing health-harming corporate practices, and challenge the notion that a primary function of NGOs can and should be to act as a ‘watchdog’ of corporate practices, instead advancing a wider perspective on corporate accountability.

## Introduction

The global production, distribution, and marketing of health-harming commodities poses a central challenge for public health. A growing body of research on the commercial determinants of health has examined the ways in which corporate actors have sought to influence policy and regulation [1, 2]. This includes the global level, where concerted efforts by companies and industry associations to obstruct the adoption of strong guidance, commitments, and binding instruments have been documented [3–5]. Yet, despite frequent appeals to the role civil society can and should play in counteracting such obstruction [6], advocacy strategies have received limited attention within research on the commercial determinants. Much like organisations representing corporate actors, non-governmental organisation (NGOs) make tactical choices about the strategies which best serve their interests [7]. In this article, we seek to illuminate the strategies NGOs employ when they *act through* global governance to influence government regulation and corporate practices. To understand this, and the decisions which underpin strategy choice, we draw on areas of scholarship with a more established focus on advocacy. Literatures on interest group behaviour, international relations, and political economy provide a rich conceptual background to understand the strategies available to health NGOs as well as the trade-offs underpinning strategic decisions. Empirically, we focus on accounts of the strategic behaviour of NGOs who engage in global governance fora with a view to promoting the regulation of health-harming industries (HHIs). We ask, *firstly*, how NGOs seek to influence regulation of ultra-processed food (UPF) and alcohol through global governance and, *secondly*, how this is shaped by the institutional context NGOs operate within. We address these questions through interviews with professional advocates from NGOs that we identified through their participation in relevant consultations and UN-led fora.

In the sections which follow, we first situate our study within existing scholarship on NGO strategy, introducing the *inside-outside* heuristic from interest group studies and discussing contributions on global civil society from other literatures. We then consider studies of NGO activity in the specific context of global HHI regulation, before turning to an examination of the governance architecture which NGOs navigate when seeking to shape the regulation of these industries.

### NGO strategy and the regulation of health-harming industry behaviours

The literature on interest groups is concerned with why and how interests organise to influence decision-making, as well as the strategies they employ and their impacts [8, 9]. A central heuristic within this area of scholarship is the inside-outside dichotomy. Traditionally applied to interest groups’ interactions with governments, inside strategies describe approaches which involve direct engagement with officials and within formal structures, such as the provision of information via consultations or meetings, whereas outside strategies encompass public-facing tactics such as protests and media campaigns [7, 10, 11]. Expanding on this classic distinction, Colli and Adriaensen broadened interest group strategies to include the market as a target of civil society advocacy [12]. A literature review by Townsend et al. applied Colli and Adriaensen’s inside-outside, state-market typology to understand the strategies NGOs use to address commercial drivers of ill-health, identifying 18 distinct strategies employed to target government regulation or commercial actors across eight industries (13; see p. 5 for a detailed list of strategies). The existing research they identify, however, is focused primarily on national and regional settings. The comparably well-studied issue areas of tobacco control and climate change suggest that global-level dynamics differ, reflecting both the greater distance of global fora from ‘publics’ which can be targeted through outside strategies and the relatively high bar for insider access which necessitates resource-pooling by civil society actors [14, 15].

Insiderness and outsiderness can be utilised to describe the status of organisations as well as their strategies. This spectrum ranges from organisations who enjoy significant access to public institutions, are recognised as authoritative by decision-makers, and primarily operate within formal processes, to those who rely largely on indirect targets like publics and the media [10]. In reality, most organisations – including NGOs – choose from a repertoire of action which encompasses diverse inside and outside strategies [11, 12, 15]. Outsiders may use outside strategies by necessity or choice, some due to a lack of direct access to decision-makers, and others based on strategic considerations or an ideological rejection of elite fora.

### Ultra-processed food and alcohol within global governance for health

The key corporate players in the UPF and alcohol industries are transnational, as are their supply chains and marketing practices. Dominant firms are largely headquartered in high-income countries, with low- and middle-income countries representing major targets for market expansion in both sectors [16, 17]. International coordination and cooperation thus present crucial mechanisms for the effective regulation of these industries. The global architectures of UPF and alcohol governance are, however, complex and dispersed. Situated at the intersection of issues like health, trade, and agricultural production, they are characterised by real and potential inconsistencies across actors, interests, and fora, as well as competition between IOs with overlapping interests [18, 19].

Following efforts in the 1990s to recognise common risk factors underlying non-communicable diseases (NCDs), the World Health Assembly (WHA) endorsed a Global Strategy for their prevention and control in 2000 [20]. The rise of political attention to NCDs was marked by a 2011 High-Level Meeting (HLM) of the UN General Assembly, the inclusion of an NCD reduction target in the Sustainable Development Goals, and the establishment of an Interagency Taskforce on NCDs [20]. Subsequent HLMs were held in 2014, 2018, and 2025. 

IOs with responsibility for food and nutrition include the Food and Agriculture Organization (FAO), the World Food Programme, and the International Fund for Agricultural Development as well as WHO and UNICEF, with the UN-Nutrition mechanism established in 2020 to coordinate inter-agency work on nutrition. Since the WHA adopted the 2004 Global Strategy on Diet, Physical Activity and Health, WHO’s focus has shifted towards supporting effective regulation of marketing, labelling, and price within member states. Although fragmented, food has long been a high-profile issue at global level. Alcohol, on the other hand, has been described as ‘global health’s blind spot’ [21], with a 2022–2030 Global Alcohol Action Plan following the limited implementation of the 2010 Global Strategy to reduce the harmful use of alcohol. Another notable forum is *Codex Alimentarius*, a joint WHO-FAO Commission recognised as a standard-setting body by the World Trade Organization and focused on the development of standards, guidelines, and codes of practice for foods and beverages [22]. Some IOs have begun work towards managing private sector engagement with a view to protecting health policy and programmes from undue commercial influence (e.g., WHO’s draft approach ‘safeguarding against possible conflicts of interest in nutrition programmes’ and UNICEF’s programme guidance on ‘engaging with the food and beverage industry’).

The emergent focus on regulation and corporate harms in the context of UPF and alcohol stands in tension with the dominance of multistakeholder norms in global governance, reflective of successful efforts by private sector actors to transform their role in governing global issues [23, 24]. The idea that issues can be effectively addressed by involving the private sector without seeking regulation is operationalised in initiatives such as the UN Global Compact, which has been encouraging voluntary commitments from businesses since 2000 [25].

## Methodology

This article draws on semi-structured interviews with policy officials, technical experts, and professional advocates that explored the policy practices and advocacy strategies of NGOs within global health. Interviewees were selected from an existing dataset of NGOs active in global health governance [26], created as part of a project exploring the regulation of alcohol and UPF industries. The dataset focuses on NGOs that are active in the global health institutional complex, specifically those that interact with the WHO, the Codex Alimentarius Commission, and the UN Global Compact, where global governance debates over UPF and alcohol are primarily situated. We adopt the definition of an NGO as ‘an independent non-profit organization that provides advocacy or other services on behalf of a constituency or group of stakeholders’ [27] (p. 232). While many NGOs engage in both service delivery and advocacy, we focus on the latter function. The term international NGO (INGO) is used here to describe NGOs who operate across borders - using simplified categories of regional vs global foci - and the term domestic NGO to describe organisations focused on one country.

Interviews followed a topic guide covering four main themes: inside and outside strategies, advocacy coalitions, perceptions and understandings of institutional contexts, and the role of policy paradigms. To ensure a diversity of perspectives, participant selection was guided by several criteria, including (a) experience and expertise; (b) demographic characteristics; (c) geographic region. In addition to an initial list of interviewees, the research also involved snowball sampling where interviewees recommended colleagues in their organisations. In total, we interviewed 22 professional advocates from 20 NGOs that engage with global UPF and/or alcohol governance. Of these, twelve had an international or global scope, five were focused regionally, and three were primarily focused nationally but active in international debates. Our analysis is centred primarily on 12 interviewees from ten INGOs who hold official relations status with the WHO and/or whose main focus is at global level. We complement this with insights from ten interviewees working at NGOs and INGOs who have engaged with insider pathways at global level, but whose primary regime is at country or regional level. Throughout the findings, we distinguish between organisations working on multiple risk factors (MRF) – for instance, on food and tobacco or food and alcohol – and a single risk factor (SRF). Two INGOs in our sample are most accurately described as humanitarian organisations. Lastly, we draw on six interviews with international civil servants employed by relevant IOs, two of whom had previously worked for INGOs. 

We analysed transcripts through an interpretive lens, sensitised to the ways in which advocates understand their role and the context within which they operate [28]. We developed a codebook through inductive coding of a subset of transcripts by the lead author, before refining it through discussion with the wider research team and integrating an existing framework by Townsend et al. [13] to form higher-order categories. In practice, this means that we adopted the distinction of NGO practices into targets (state vs. market) and strategies (inside vs. outside) – as originally proposed by Colli and Adriaensen [12] – and coded to specific strategies identified by Townsend et al. [13] where relevant/applicable. We further draw on Townsend et al.’s distinction between substantive, procedural, and normative impacts to understand advocacy aims and impacts. Impacts were considered *substantive* if they entailed changes to policy or corporate practices. *Procedural* impacts, on the other hand, encompass changes to decision-making processes and representation within. Lastly, *normative* impacts reflect changes in how issues and actors are framed and understood.

## Findings

### NGO strategies to regulate health-harming commodities through the global level

#### Targeting IOs and member states

For globally focused health INGOs, engagement with the UN system is centred on WHO. UNICEF, UNDP, and UN-wide NCD and nutrition agendas provide additional entry points within global health governance, while several interviewees noted efforts to expand into fora like Codex Alimentarius. NGOs discussed their use of a breadth of strategies: they primarily target IOs directly through participating in consultations, writing and signing open letters, holding bilateral meetings with international civil servants, and leveraging WHO official relations status. Importantly, tactics are often interdependent, with written consultation responses, for instance, also functioning as public position papers and forming a basis for engagement with IO officials. Much of this engagement pursues the ultimate aim of influencing member states, who are key targets in multilateral spaces. As such, lobbying of country missions and representatives is a routine aspect for many NGOs operating at the global level. One participant illustrates the interconnectedness of these activities:

*the way we would engage is essentially*,* we do our own primary research*,* collecting evidence that we make available to WHO or to parties via WHO. We comment on documents*,* we provide submissions*,* official submissions. Essentially*,* we advocate through official routes online … And to the extent that we can*,* we will participate in advisory committees or other forms in which WHO seeks this kind of input. *(global INGO, MRF)

Common outside strategies such as the use of social and traditional media, or the publication of reports or position statements were, in most cases, described as primarily targeting IO decision-making. Though the importance of increasing public support for action on UPF and alcohol was often highlighted, this was not positioned as a key step towards influencing global governance. Further, public-facing boycotts do not appear to be part of the repertoire of tactics employed by these INGOs to shape UPF and alcohol regulation globally, though a small number of interviewees note making strategic decisions to withdraw from multistakeholder spaces, or leveraging potential withdrawal to influence IO officials.

Interviewees from domestically and regionally focused NGOs presented a more ad-hoc approach to global engagement, centred on *‘big moments like [the] World Health Assembly’* (regional INGO, MRF) and more directly targeted at supporting domestic advocacy, for instance, by bolstering legitimacy through association with IOs or by working towards stronger international guidance which can be mobilised domestically.

### Targeting the market

Most NGO interviewees did not include direct targeting of or engagement with corporations as a routine component of their advocacy. Monitoring and reporting on commercial practices, classified by Townsend et al. [13] as an outside strategy targeting the market, was considered by interviewees primarily as a mechanism to encourage public accountability via governments or IOs, rather than a tool to directly challenge and change corporate behaviour. As one advocate explained,

*the monitoring and accountability side of things is as much about strengthening the evidence base for governments to act more robustly because NGOs themselves obviously can’t regulate the industry. But it’s about building knowledge of practices that can then help governments to take the right decisions around what they need to be doing to better regulate industry. *(global INGO, MRF)

Notably, although participants recognised the importance of independent monitoring of corporate practices, they challenged the idea that being an industry watchdog can or should be a primary function of global civil society. One advocate articulated a widely shared sentiment in arguing that *‘even though we like the watchdog role*,* there is no way that we can be there*,* all over the place. But we can certainly do spot monitoring to prove a point’* (global INGO, SRF).

### Engaging beyond WHO

While interviewees shared a recognition that engagement with fora beyond WHO is important and necessary, such interactions remain largely ad-hoc and limited. Advocates most commonly noted the UN Food Systems Summit (UNFSS) and Codex Alimentarius as relevant fora, with a few describing efforts to engage with UN climate negotiations. The World Bank, UNDP, and UNICEF were noted by three or four interviewees each as institutional targets, whereas FAO was recognised as powerful but challenging to engage with. The disconnect between the importance assigned to engaging beyond health and the limited nature of actual engagement is explained primarily through reference to resource constraints which prohibit sustained engagement and thus the establishment of authority in new spaces:

*As health NGOs we have the WHO … and then you go to FAO you realise OK*,* now there’s another whole group of us*,* we’re just at this other UN institution*,* and we’re new coming into this conversation*,* how do we get heard amongst these other interests? So that’s usually the challenge when you try to engage. We’ve engaged with WIPO*,* we’ve tried to engage with WTO etcetera*,* but you’re an outsider to some extent. *(global INGO, MRF)

One reason engaging with new fora poses such a resource challenge is the procedural and substantive knowledge it requires. Many INGOs, for example, do not participate in the Codex Alimentarius Commission process despite recognising its crucial role as a potential impediment or facilitator of health policy action. Only two INGOs in our sample were Codex observers at the time of interview, and they highlighted persistent barriers to full participation. One advocate whose INGO is a Codex Alimentarius observer pinpoints key difficulties as

*knowing which [Codex Alimentarius updates] are relevant*,* which ones you should track*,* it’s just literally a staffing issue actually. I think if one had the bandwidth to study it and spend time on it*,* one could figure it out. But it takes a lot of time to get familiar and comfortable with what they’re up to. And I think this is the constraint in non-profits*,* we don’t have an army of people to represent us in all of these spaces. *(global INGO, MRF)

Further, differing norms regarding private sector engagement outside the global health regime were invoked as barriers to successful expansion by health NGOs. In the context of the FAO, for instance, one advocate observed that engagement on regulating UPF is more challenging because the organisation’s *‘main private sector stakeholder is [the] food industry’*. These tensions are at the core of discussions about the UNFSS, a process widely contested for its failure to problematise corporate power and to ensure equitable civil society representation [29, 30]. Concerns about the implications of a strong industry presence and emphasis on partnership for meaningful civil society involvement underpinned decisions to disengage by NGOs within our sample. One advocate, for example, explained that their organisation decided not to participate *‘because we believed that it would be completely overrun by industry’*, noting that *‘from a resource constraint issue*,* it felt like it was not a good use of our time and resources*,* that it was already in some ways fixed’* (global INGO, MRF).

### Advocacy objectives

Advocates pursued an interconnected set of substantive, procedural, and normative goals within global governance. **Substantively**, a primary goal of NGO advocacy directed at the WHO is to support the development and publication of strong guidance and strategies. This includes guidance on NCD prevention, nutrition, and alcohol policy, such as the SAFER initiative on alcohol harm or the set of ‘Best Buys’ to address NCDs. Maintaining the issue of alcohol harm on relevant global health agendas emerged as a specific goal, with the previous absence of alcohol as a risk factor from the UN NCD agenda described as *‘an illustration of how alcohol often falls out of context where it naturally belongs’* (global INGO, SRF). Interviewees recognised the capacity of global, binding agreements to not only facilitate substantive policy progress, but also rally civil society and provide clear structures for ongoing engagement. One participant, for instance, looked towards climate governance, observing that, in the context of UNFCCC, civil society coalitions have benefitted from *‘over time having a platform where they have built up a way of working’* (global INGO, MRF). The FCTC – and particularly Article 5.3 which requires parties to protect tobacco control policies from tobacco industry interference – was most commonly invoked as a potential model for governing UPF or alcohol, though this prospect met with differing levels of enthusiasm and optimism. Interviewees conveyed a sense that, for food and alcohol, a global agreement would be significantly harder to achieve than it had been for tobacco over two decades ago:

*the FCTC was definitely a landmark and I think one of the interesting things is some of the principles that [it has] enshrined. Particularly [Article] 5.3 in terms of non-engagement … I don’t know to what extent this is because it’s tobacco control and the actors are slightly different versus alcohol control and the way that alcohol is kind of embedded in particular within Europe*,* North America*,* Australia and New Zealand*,* but it’s sometimes quite hard to encourage member states to transfer principles. *(global INGO, MRF)

Estimating that we are *‘a decade away’* from the level of evidence required to move towards an internationally binding agreement on alcohol, one advocate explained that when looking *‘at the countries and … at the evidence and where people stand currently in their statements*,* it feels like it would be a heavy lift to get anything more substantial’* (global INGO, MRF). This sentiment is echoed implicitly by another global advocate who noted that, while the long-term objective of achieving a globally binding agreement for alcohol *‘is the basis of everything’* their INGO does, their work remains centred on more immediate issues and opportunities (global INGO, SRF).

**Procedurally**, improving safeguarding against commercial influence is a central ambition across NGOs seeking to regulate UPF and alcohol industries. Advocates are working to limit the engagement of WHO and other multilateral bodies with UPF and alcohol industry actors, with a view to minimising corporate influence on decision-making. Procedural changes pursued by participants further include greater representation of civil society in general and of underrepresented regions and groups more specifically.

As regards **normative** goals, participants emphasised the need to shift collective understandings of how to address NCDs away from individual responsibility towards a focus on environments and the role of commercial actors in shaping them. Specifically – and related to procedural goals discussed above – efforts to denormalise the involvement of UPF and alcohol industries in policymaking, and multistakeholderism as a mode of governance which enables this, are central pillars of participants’ efforts to promote effective regulation of HHCs. In so doing, they recognise the normative power institutions such as the WHO can exert in setting strong guidance on private sector engagement.

As part of a strategic aim to raise awareness of industry interference and conflicts of interest, some NGOs also target or engage with other civil society actors. One advocate working across risk factors describes how their organisation

*[does] a lot within the civil society community itself. To both raise awareness of these issues around conflict of interest*,* industry interference but also to try and promote solutions and ways of responding and that we’re all singing from the same song sheet as it were. *(global INGO, MRF)

### The technocratic politics of intergovernmental institutions

Most interviewees articulated a sense that non-confrontational, inside advocacy in global health governance is not only more effective but also more context-appropriate. One interviewee noted that while there is a *‘kind of a protective side of global [advocacy] organisations being able to be noisier than potentially national ones’*, they see IOs as less responsive to noisy, outside politics (global INGO, MRF). This was connected by some participants to an assumption that officials and institutions with a health mandate share the same overarching goals and INGOs’ role is thus primarily to support and empower them. Consequently, targeting policymakers in global fora through noisy, public-facing campaigns is seen as a potential risk to relationships.

Several INGO interviewees signalled awareness of their capacity to bolster the position of WHO, illustrated by a reflection that *‘WHO would like to see more statements from NGOs and non-state actors [at WHA] because the statements are a way of putting a bit of pressure to the member states on delivering the resolution’* (global INGO, SRF). This highlights a less radical outside strategy commonly discussed by participants. Similarly, INGOs with insider relationships with IO officials can support advocacy within or between institutions. This may take the shape of information provision behind the scenes or noisy mobilisation of outside pressure to legitimise proposals, hold IOs to account, or overcome perceived blockages. One advocate and an international civil servant discussed an example where insider relationships across IOs and civil society were mobilised to advance shared objectives; in this instance, officers within a UN agency encouraged a civil society report challenging its partnership with a harmful commodity company, and subsequently utilised it internally to advocate for changes to the agency’s practices.

Others, however, invoked institutional norms, with a small subset of interviewees reflecting critically upon the relatively quiet nature of global advocacy on HHIs and contrasting these fields with women’s health, climate change, and human rights where noisier tactics were seen to be more commonplace. One advocate, for instance, described the WHO as a *‘European’* forum where *‘[t]here are formal ways to engage and people stick to these formalities’*. Arguing that UPF *‘warrants the same level of rage’* as traditionally noisy issue areas like human rights, they go on to contemplate that the latter may be *‘better organised and better funded to organise these things [such as rallies and protests]’* and to speculate that *‘maybe what [health] is missing is that enemy that you can see’* which environmentalists have in polluters (global INGO, SRF). Several interviewees from regional and domestic NGOs voiced frustration for similar reasons. One advocate from the Global South explained their perception of WHO officials as *‘generally very bureaucratic. You have to follow the system and if you don’t follow the system*,* that is just not going to go anywhere’* (regional INGO, SRF). They proceed to argue that a shift towards more ‘outside’ advocacy within global health governance is necessary to counteract the extent to which NGO engagement is currently structured – and constrained – by nature of WHO as a forum: *‘I think as NGOs we need to own a different space’* (regional INGO, SRF). Conversely, an experienced advocate argued that, owing to a lack of alternatives, NGOs engage *‘by default’* with UN-led fora, even though some may

*see WHO Geneva as colonising almost because then when you’re down on the ground*,* people are saying like these things are being decided in Geneva and it’s just not the way that Latin America works*,* it’s not the way Africa works. How many Latin Americans or Africans do you actually have in Geneva? *(global INGO, SRF)

The overall preference for quiet politics among respondents is also reflected in the response of health and nutrition groups to fora like the UNFSS. Though INGO interviewees shared disappointment with the Summit, those who disengaged from the process did so gradually and partially, which stands in contrast to the boycott and counter-summit organised by the food sovereignty movement [30]. Although they identified a clear risk in the Summit’s embrace of private sector collaboration, INGOs’ decisions to remain engaged appeared rooted in the perceived potential for influence over member states and IOs promised by high-level engagement. An interviewee from a globally focused INGO explained that *‘even when you find out industry is deeply embedded*,* well*,* it’s UN-organised so you have to be there to make sure*,* you have to be there to raise the voice at member states present*,* “Why have you let them in?”’*. Another advocate’s rationale for ongoing engagement with the Summit process was the heightened political priority member states assigned to this forum, which opens distinctive opportunities to access high-level political representatives:

*It is crazy also to see when you go to the [UNFSS]*,* for instance this year’s [2023] Stocktaking Moment*,* the amount of high-level prime ministers*,* presidents that are attending*,* versus the World Health Assembly just gets a video from a few prime ministers and that’s it. My point here is that health within government is often so under-prioritised*,* especially at the global level. *(global INGO, MRF)

### The quiet modalities of outside strategies at global level

Outside strategies were positioned by most interviewees as an option to be deployed only in the case of blockages or failure of quieter, inside approaches. Though INGO advocates noted that they do utilise tactics such as media engagement to challenge global health actors and institutions with regard to the role of HHIs in global governance, there was a clear preference for conveying such messages in direct engagement with policymakers or through reports, briefings, and open letters. One advocate explains that their organisation *‘[starts] with the quieter diplomacy route and if we realise we’re not getting anywhere or we can’t do anything*,* we’re not seeing the outcome that we want*,* then [we] quite often shift to the more noisy approach’*, illustrated using the example of public statements (global INGO, MRF). This represents the use of an outside tactic as something of a last resort, commonly coordinated collaboratively through NGO networks to maximise attention and impact. Such publications often challenge the role of industry in policy and governance: examples mentioned in interviews include open letters to IOs about dialogues or partnerships with HHI actors, and reports critiquing specific multistakeholder initiatives.

This hesitancy to disrupt too outright is illustrated by one advocate who recounted opting for a public statement to express discontent with one of several recommendations from a government-led multistakeholder advisory body to WHO, because they felt that *‘if I was to completely remove myself from the [advisory body] that would be quite risky but we needed to make a clear statement that we weren’t agreeing with that specific part of the report’* (global INGO, MRF). This signals a greater willingness to consider employing more confrontational approaches when targeting commercial actors, public-private partnerships, or even IOs where the insider route is unavailable. One advocate, for instance, argues that their organisation *‘would obviously be noisier around actual actions by industry’*, although *‘the goal for that is always to get regulation of those practices’* rather than influencing companies directly (global INGO, MRF).

More generally, participants shared an acknowledgement of the tension between insider NGOs’ need and desire to maintain long-term, supportive relationships with global policymakers, and their aspiration to hold them to account. *‘[Y]ou want to maintain that good relationship but you also want to be able to be critical when the time is required’*, maintains one interviewee; a balance which can prove *‘a bit tricky’* (global INGO, MRF). This is echoed by another advocate who referenced *‘controversial discussions where WHO is not happy with us [advocates]’*, noting that while this renders joint working more fraught, *‘WHO also has to be accountable and that’s our job’* (global INGO, SRF). One domestic advocate experienced in engaging with global governance criticised that *‘sometimes advocacy campaigners can direct their guns at WHO themselves for – in their words – kind of mishandling the industry and not excluding the industry’*, warning that this

*can be a real risk to us*,* because they’re the key policy actors that we want to win over and that can deliver things for us. So I’m not sure that being hyper-critical of WHO is necessarily wise*,* and also fair. … we need to be realistic about where their resources come from*,* where their political direction comes from. *(domestic NGO, SRF)

### Gaining and maintaining insider status

Continuous investment of resources is necessary to uphold relationships, maintain legitimacy and credibility, and understand and adapt to norms within a regime. Engagement with the WHO primarily focuses on the World Health Assembly (WHA) and Executive Board sessions, and related processes connected to specific strategies such as developing the Global Alcohol Action Plan. While coordination around these governing body meetings demands significant time and resources, they are seen as crucial touchpoints for global health advocacy. As one interviewee notes, *‘these high-level moments really do kind of give us direction’* and *‘convene us around an issue to carry it forward that way’* (global INGO, MRF). For many NGOs, engaging effectively with high-level events requires resource-pooling with partners. Not all INGOs, for instance, have capacity for sustained outreach to Permanent Missions, especially considering the long-term relationship building and intelligence gathering on evolving policy positions this requires. An advocate from a smaller INGO without a Geneva base, for instance, explains that with very few staff members focused on policy, *‘the capacity to engage regularly and reliably with missions isn’t quite there*,* so that is something that we rely on larger organisations … to do’* (global INGO, MRF).

Preparation for events like WHA and the Executive Board begins months prior, and involves aligning positions and messaging with partner NGOs, developing statements, and briefing Permanent Missions. As one interviewee reflects, *‘for these big convening moments*,* it’s all done before we get there and then once we get there it’s just the presentation of it’* (global INGO, MRF). For INGOs operating as insiders within global health, obtaining and maintaining this status entails substantive costs. NGOs can send delegates to official meetings and make statements only if they are in official relations with the WHO: a privileged status the Executive Board may grant international non-state actors based on sustained, systematic engagement with the institution [31]. The process of obtaining official relations was described as complex, but while the status is not necessary to interact with WHO, most of the globally focused organisations in our study had sought it out to strengthen engagement and gain the right to deliver statements at WHA.

While relatively open in principle, the WHO’s formalised engagement processes are, in practice, challenging to access for many. Only international organisations are eligible for official relations status, though domestic NGOs can and do nonetheless engage with the WHO through meetings and consultations. Partner INGOs can also facilitate access and representation, for instance, by highlighting opportunities for input, offering space on their delegations, or sharing allocated speaking slots. Perhaps unsurprisingly, participants with domestic or regional foci described closer relations with regional WHO offices, which are not only more proximate but also seen as more open to civil society.

### Institutional and political influences on NGO strategy choice

As discussed above, the institutional and political environment provided by the institutional architecture NGOs routinely engage with was perceived as more receptive to an overall quiet approach to advocacy. Yet, this preference was also echoed in the sense of organisational identity interviewees conveyed, not without some frustration:

*[some of our board members] prefer the diplomacy routes*,* the quieter approach to advocacy. So we’re obviously not a Greenpeace where we’re going to suddenly be doing direct actions around the world or anything like that*,* or creating*,* organising marches and protests*,* even though I’d like to. It’s not [us]. [My INGO has] tended to focus more on the quiet side and our noisy is probably quite tame compared to a lot of NGOs. But we recognise that there’s always a need for a spectrum of different organisations with different tactics and strategies and there’s many other organisations in the NCD space that are much more on the noisy level and that works well because we do the more diplomacy stuff. *(global INGO, MRF)

Another interviewee from the same organisation noted that *‘we are not rock chuckers by nature’* but instead seek to *‘influence within a process before we go externally’*, noting the clear difference between *‘making statements or … publishing a criticism versus much stronger campaign tactics’* (global INGO, MRF).

Participants further conveyed a clear sense that advocacy across different HHIs, though often aligned in demands, can face different challenges and thus must adapt strategically. Interviewees noted the limited applicability of strategies that succeeded in tobacco control, recognising the absence of global coordination around clear goals as well as differences in perceptions of product harm. The lack of pre-existing infrastructure for the regulation of alcohol, relative to more established work on food safety and nutrition, means that alcohol advocates are seen by some as facing a greater challenge, with one interviewee arguing that the case for alcohol regulation needs to be built *‘from scratch’* (global INGO, MRF). One advocate working across risk factors noted that their INGO’s positions on alcohol, UPF, and tobacco differ: *‘there is no wording around endgame with alcohol as there is for tobacco*,* but both tobacco and alcohol are products that we can live without’*, whereas *‘food is a bit trickier*,* because it’s essential for living*,* and a lot of the big corporations selling food also to some extent sell food that is healthy and necessary for nourishing the population’*. They further reflected that another reason food is *‘trickier to navigate than alcohol and tobacco [is that] there is this red flag’* around working with industry in the latter cases. These concerns are echoed in another interviewee’s description of *‘alcohol [as] a luxury*,* food [as] an essential’*, and associated concerns about inadvertently exacerbating issues of undernutrition underpinned a more hesitant approach towards food in their global, cross-risk factor INGO.

## Discussion

In this article, we set out to understand the strategies NGOs use to influence the global governance of UPF and alcohol, and the strategic choices which underpin them. Alongside substantive goals to ensure strong guidance and recommendations on alcohol and nutrition policy, we identified shifting norms around private sector engagement and *‘crafting’* new spaces for the regulation of HHIs as key advocacy aims. There is a clear recognition of the power of regime-building in the context of the FCTC and the Code of Marketing of Breastmilk Substitutes, both substantively and as a focal point for advocacy activities. Notwithstanding some desire to work towards legal frameworks for food and alcohol, views on feasibility and political palatability diverged among the professional advocates we interviewed. Interviewees reported that their organisations pursue these goals primarily by advocating for government regulation rather than engaging directly with industry. This reflects an understanding of the limited potential of transnational private regulation to meaningfully contribute to achieving health and sustainability goals [12, 32, 33]. Correspondingly, emphasis was placed on contesting the dominance of multistakeholderism, challenging the status quo of uncritical engagement with private sector actors, and fostering alternative spaces where industry interactions are tightly circumscribed.

Interviewees from the Global South, however, echoed critiques of global public policy as an elite project [34] and challenged the continued predominance of INGOs from wealthy countries within global health governance. What emerged as a key constraint for INGOs with the capacity to act as insiders within the relevant fora is a sense that the range of tactics available are narrowed by what is *appropriate*. In practice, this translated into a preference for less confrontational approaches. In addition to the financial costs of sustained participation in global fora (including staff time for monitoring, preparation, and attendance, as well as travel), the institutions of global health governance remain inaccessible for many. The structural barriers to entering and maintaining engagement with global fora are illustrated by the criteria for accreditations like WHO official relations or Codex Alimentarius observer status. Both are limited to INGOs and require an established headquarters, constitution, and governing body, as well as sustained activity for several years [31, 35]. In practice, this means that they are difficult to access for more informally organised movements, as well as nationally or regionally focused NGOs. In the absence of structural change, coalitions are crucial for mitigating such barriers, with INGOs who hold observer status commonly enabling others’ participation by sharing places on their delegation.

Where interviewees reflected on differences between the issue areas of food and alcohol, regulating the latter was seen as more politically feasible in broad terms, while the more developed governance infrastructure for food was seen to provide a stronger starting point. Further, the centrality of the WHO – its unrivalled competence in a given issue area [36] – is greater for tobacco and alcohol than for food, where the FAO holds significant authority. The UNFSS, for instance, saw the WHO and the wider health community occupy a relatively marginal position. Perhaps connected to the more diffuse nature of global food governance is the *‘lack of a specific shared goal’* that Friel [37] identifies as a core issue for the healthy and sustainable food systems movement. Interviewees noted fragmentation even within the dietary public health space, between groups working primarily on undernutrition and those working on overconsumption, who often diverge in their views about the role of the food industry in health governance. Crucially, greater engagement beyond health fora was highlighted as necessary to effectively addressing health-harming industries, but challenging to achieve primarily due to capacity constraints.

More broadly, the rise of multistakeholderism as a governance norm and the implications of this shift for corporate power were seen as central challenges for global advocacy on UPF and alcohol. Though not explicitly framed in these terms, much of the advocacy explored in this article is centred around an expansive conception of corporate accountability; engaging with the rich scholarship on this function of advocacy may thus offer new ways of understanding the role of NGOs in the global governance of HHIs. Newell [38] advances an understanding of corporate accountability as not limited to direct efforts to influence corporations and their representatives, but also comprising indirect accountability via government policy: *civil regulation* and *public accountability*, respectively. The organisations in our study primarily pursue the latter route. Although several engage in industry monitoring, they challenged the idea that this can or should be a core component of their advocacy, which aligns with existing evidence that monitoring and enforcement of corporate activities is a relatively less common governance function for NGOs active within the global governance of UPF and alcohol [39]. This calls for a recasting of the pathways and targets involved in achieving corporate accountability beyond the ‘watchdog’ model so often articulated in global health.

Global health has seen protests and boycotts work, but in the governance of HHIs today there is little resembling, for instance, the strategies advocates used in the 1970s to challenge the aggressive marketing of breastmilk substitutes [40]. This is reflected in interviewees’ emphasis on inside strategies and information-based outside strategies as more appropriate and effective in global fora concerned with UPF and alcohol regulation. A perceived norm which inhibits noisier, more confrontational behaviour by NGOs echoes well-documented pressures that discourage protest tactics within global governance [41]. It also means that the use of such strategies presents a perceived risk for NGOs seeking to establish or maintain authority. Stroup and Wong [42] describe this trade-off as the *‘authority trap’*. Starting from the recognition that the vast majority of NGOs will remain in obscurity, they argue that the few organisations who succeed in establishing themselves as *‘leading NGOs’* find their strategic choices constrained by the need to maintain this status. Our findings align with Stroup and Wong’s wider argument in indicating a reluctance by leading INGOs to condemn the governments or IOs they routinely engage with, and a focus on more incremental *‘vanilla victories’* instead of more radical change.

To better understand NGO strategy and impacts, future research could trace how advocacy evolves over time, and in response to changing political and governance environments. Some have observed that blockages and delays in government and IO action on environmental issues have prompted shifts within civil society towards greater bottom-up, outside pressure or increased direct engagement with TNCs [38, 43]. This shift does not – or not yet – seem to be occurring in the context of UPF and alcohol, where NGOs are pushing for more incremental action and engaging constructively with a UN agency that is largely sympathetic but lacking in power. Further exploration of shifts in NGO structure, identity, and strategy alongside, and in response to, changing institutional architecture would also be valuable. The International Baby Food Action Network, for example, started off as a loose coalition focused on protest tactics, but now corresponds more closely to the idea of an insider group, with WHO official relations status and a key role in monitoring compliance with the Code of Marketing of Breast-milk Substitutes. Another NGO instrumental in the Nestlé boycott, Infact, instead broadened their campaigning under the name Corporate Accountability International, maintaining a greater focus on civil regulation via public pressure on TNCs, accompanied by efforts to strengthen democratic and multilateral institutions [44].

### Limitations

This study is focused on exploring the strategies used by formally established NGOs who operate primarily as insiders within global health governance and advocate on UPF and alcohol regulation. As such, the findings presented here only offer insights into the strategic decisions made by this sub-group of civil society actors and the institutional foci they adopt; NGOs with a main or sole focus on direct engagement with industry (e.g., via shareholder activism or partnership initiatives), movements whose express aim is to challenge global health structures from the outside, and individual activists, are outside the scope of this analysis. Moreover, our analysis did not aim to quantify the relative prevalence of different strategies and necessarily reflects the views and experience of a subset of professional advocates.

## Conclusions

Global advocacy for UPF and alcohol regulation requires NGOs to navigate complex architectures which, even where ‘open’ in theory, are often challenging to access in practice. This reinforces the centrality of a small number of INGOs from the Global North who operate with a great degree of authority and access; a status which conversely implies self-imposed constraints on their range of action. As a result, INGO strategies aiming to hold UPF and alcohol industry actors to account at global level primarily take a quieter, inside approach centred on the WHO and member states. Recognising successes of campaigns such as those on infant formula marketing, the *Lancet* series on commercial determinants of health encourages civil society to *‘be noisy’* and make the case for action on harmful commercial practices not only to governments but also to communities [6]. This is perhaps even more pertinent when entering trade, food systems, or climate governance spaces, where many health ‘insiders’ operate without the authoritative status which inhibits their range of action. The importance of engaging beyond the health domain is well-recognised, but capacity constraints mean that many are not able to make the long-term resource investment required to enter and establish authority in a new forum. Considering the new leverage points and access to high-level decision-makers participating in fora such as Codex Alimentarius enables, there is a clear need for enhanced support from advocacy funders for strategic engagement beyond health as a key pillar of advocacy to address commercially driven harms.

WHO and PAHO staff, writing in an edited volume about NCDs [20], suggest that progress towards translating NCD-related commitments into action has been slow partly due to *‘the lack of a sufficiently strong civil society voice calling for accountability for the commitments made when for example compared to the way that civil society advocated for action on AIDS*,* TB and malaria’* (p. 233). While challenges around funding for and public campaigning on NCDs are well-recognised, the role of corporate power in upholding the status quo must be acknowledged as a crucial factor shaping the global governance HHIs. Our findings reflect a perception by professional advocates that the structural power of corporate actors within global governance remains a, if not the, core challenge for NGOs. Correspondingly, there is much research and advocacy in this field can gain from engaging with other areas where corporate interests stand at odds with health or environmental protection – notably the recognition that corporate accountability is not the responsibility of civil society alone.

## Data Availability

The datasets generated and analysed during the current study are not publicly available due to a high risk of participant identification.
